# A Truncation Variant of the Cation Channel P2RX5 Is Upregulated during T Cell Activation

**DOI:** 10.1371/journal.pone.0104692

**Published:** 2014-09-02

**Authors:** Pierre Abramowski, Christoph Ogrodowczyk, Roland Martin, Olaf Pongs

**Affiliations:** 1 Institute for Neuroimmunology and Clinical Multiple Sclerosis Research (inims), ZMNH, University Medical Center Hamburg-Eppendorf, Hamburg, Germany; 2 Research Department Cell and Gene Therapy, Clinic for Stem Cell Transplantation, University Medical Center Hamburg-Eppendorf, Hamburg, Germany; 3 Institute for Neural Signaltransduction, ZMNH, University Medical Center Hamburg-Eppendorf, Hamburg, Germany; 4 Neuroimmunology and MS Research, Department of Neurology, University Hospital Zurich, Zurich, Switzerland; 5 Institute for Physiology, University Hospital Homburg, Homburg/Saar, Germany; New York University, United States of America

## Abstract

Members of the P2X family of ligand-gated cation channels (P2RX) are expressed by various cell types including neurons, smooth- and cardiac muscle cells, and leukocytes. The channels mediate signalling in response to extracellular ATP. Seven subunit isoforms (P2RX1-P2RX7) have been identified and these can assemble as homo- and heterotrimeric molecules. In humans, P2RX5 exists as a natural deletion mutant lacking amino acids 328–349 of exon 10, which are part of transmembrane (TM) 2 and pre-TM2 regions in other organisms like rat, chicken and zebrafish. We show that P2RX5 gene expression of human T lymphocytes is upregulated during activation. P2RX5 is recruited to the cell surface. P2RX5-siRNA-transfected CD4^+^ T cells produced twofold more IL-10 than controls. Surface and intracellular P2RX5 expression was upregulated in activated antigen-specific CD4^+^ T cell clones. These data indicate a functional role of the human P2RX5 splice variant in T cell activation and immunoregulation.

## Introduction

An intimate cell-cell contact between a T cell and an antigen-presenting cell (APC) elicits T cell activation. It is associated with immunological synapse (IS) formation at the T cell surface, morphologically recognizable as a polarized structure, supramolecular activation cluster (SMAC) [1]–[3]. Detailed immunological studies have investigated and characterized the role of SMAC proteins in the initiation process of IS formation [1], [2]. Much less is known about later phases of T cell activation, involving IS organization and maintenance [4]. CD4^+^ T cell interactions with APCs at the IS may last for 6 h or more [5], [6]. IS-engagement results in Ca^2+^-mediated signalling events which participate in modulating T cell activation [7]–[9]. Depending on its timing and composition IS formation may result in several outcomes including anergy induction, full activation, activation-induced cell death, and these are involved in tight control of T cell activation under physiological and autoimmune conditions [10]–[13].

To mount an efficient immune response activated T cells require a sustained increase in intracellular Ca^2+^ concentration [Ca^2+^]_i_ preceded by elevated Ca^2+^-ion influx [14]–[17]. This involves upregulation of ion channels, such as the Ca^2+^ release-activated Ca^2+^ channel (CRAC) and the Ca^2+^-activated potassium intermediate/small conductance calcium-activated channel, subfamily N, member 4 (KCNN4) K^+^ channel, which accumulate within the IS at the cell surface of the activated T cell [18], [19]. As an early step of the activation process ion channel mRNA expression is upregulated resulting in increased ion channel density at the cell surface. Here, we wanted to address if T cell activation involves upregulation of additional ion channel activities to effectively regulate [Ca^2+^]_i_ homeostasis and to clamp elevated [Ca^2+^]_i_ for longer durations. Therefore, we activated primary human CD4^+^ T cells and systematically characterized changes in expression levels of ion channel mRNAs by using oligonucleotide-based arrays. In addition to CRAC and KCNN4 channel subunits, T cell activation affected expression levels of only a few other ion channel mRNAs. The most prominent mRNA upregulation, however, was observed for purinergic receptor P2X, ligand-gated ion channel, 5 (P2RX5), a member of the purinergic receptor gene family 2 with unknown function in humans [20]. We show that P2RX5 accumulates at the surface of activated CD4^+^ T cells. Moreover, both intracellular and surface expression of P2RX5 by human T cell clones (TCCs) were dependent on T cell activation. P2RX5 mRNA knock-down experiments established P2RX5 as a novel regulatory component of T cell polarity and implicate P2RX5 in the regulation of synaptic IL-10 secretion. Hence, P2RX5 represents a functional surface membrane component of activated T cells with an apparent role during the later phase of T cell polarity and the secretion of the regulatory cytokine IL-10.

## Results

### P2RX5 is upregulated during CD4^+^ T cell activation

**Figure 1:**
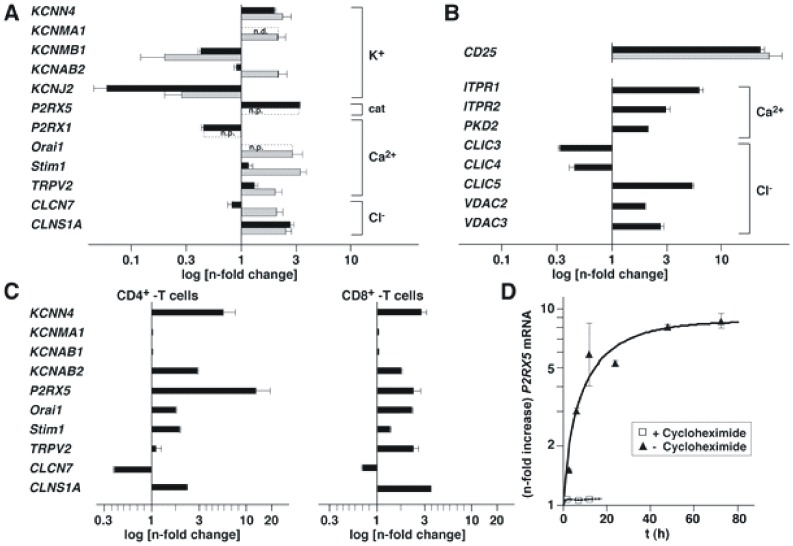
T cell activation alters mRNA expression of specific ion channel subunits. A, B, Bar diagram summarizing n-fold increase or decrease in intensity of hybridization signals obtained by probing oligonucleotide-based arrays with Cy3 and Cy5 labeled cDNA derived from mRNA isolated from non-activated and PHA-L activated PBMCs, respectively. Gray bars – custom-made array (n = 14) (Tables S1, S2); black bars – Affimetrix array (human U133A 2.0; n = 6). Error bars are SEM. Channel subunit genes are indicated on the left. Nomenclature is from http://www.ncbi.nlm.nih.gov/omim. Brackets - selectivity of corresponding ion channel. cat – cation; n.p. – not present in array; n.d. – not determined. CD25 (IL2RA) served as control. C, Bar diagram summarizing qPCR results for changes in mRNA expression of purified CD4^+^ and CD8^+^ T cells activated with anti-CD3/CD28 antibody-coated beads (n = 5–8). D, qPCR analysis of P2RX5 mRNA expression in activated CD4^+^ T cells in absence (▴) or presence (□) of cycloheximide (n = 3). Error bars are SEM.

In exploratory experiments we stimulated PBMCs with PHA-L for 72 h to profile changes in mRNA expression of 188 subunits of cell surface ion channels with a custom-made oligonucleotide-based array (Table S1). Activation of primary human T cells resulted in a ≥ twofold increase or decrease in mRNA expression (Fig. 1A, B; Table S2) of only a few ion channel subunit genes (upregulated: TRPV2, KCNAB2, KCNMA1, KCNN4, CLCN7, CLNS1A, STIM1, Orai1; downregulated: KCNJ2, KCNMB1). This compares with a twenty-six-fold increase in expression of CD25 mRNA, a prototypic marker for T cell activation (Fig. 1B). Subsequent analysis using a genome-wide expression array, which extends the above experiment to ion channel subunits targeted to intracellular compartments (Fig. 1B), indicated a comparably small number of ion channel subunit genes that displayed a ≥ two-fold increase or decrease in expression upon PHA-L stimulation (Fig. 1). Consistent with our hypothesis, PHA-L activation of PBMCs upregulated a distinct mRNA set for ion channels, e.g. IP_3_ receptors, Ca^2+^-regulated Ca^2+^-, Ca^2+^-activated K^+^-, and Cl^-^ (anion)-channels. Recent data on transient receptor potential (TRP) expression in activated primary human T cells describe increased expression of TRPM2 96 h after activation with anti-CD3/anti-CD28 antibody-coated beads [21]. Notably, the additionally found TRPC3 upregulation, which shows different kinetics compared to TRPM2, was not identified by our genome-wide expression array.

We then quantified mRNA expression of specific cell surface ion channels in activated T cells. We stimulated purifed CD4^+^ and CD8^+^ T cells with PHA-L or with soluble anti-CD3 before separating the T cells for qPCR analysis (Fig. S1A, B). Subsequently, we analyzed mRNA expression of purified CD4^+^ or CD8^+^ T cells separately following activation with anti-CD3/anti-CD28 antibody-coated beads (Fig. 1C). The different stimulation protocols yielded qualitatively similar results. In comparison to the array data, the qPCR data, however, exhibited markedly larger changes in mRNA expression (Fig. 1C; Fig. S1A, B), in particular for the expression of KCNN4 and P2RX5 mRNA in CD4^+^ T cells (Fig. 1C). The ∼ thirteen-fold increase in the level of P2RX5 mRNA in activated CD4^+^ T cells (13.0±1.2; n = 3) stood out against data of the other ion channel subunit mRNAs. Therefore, we concentrated in our further investigations on P2RX5, which is highly expressed in lymphoid tissue [22].

**Figure 2:**
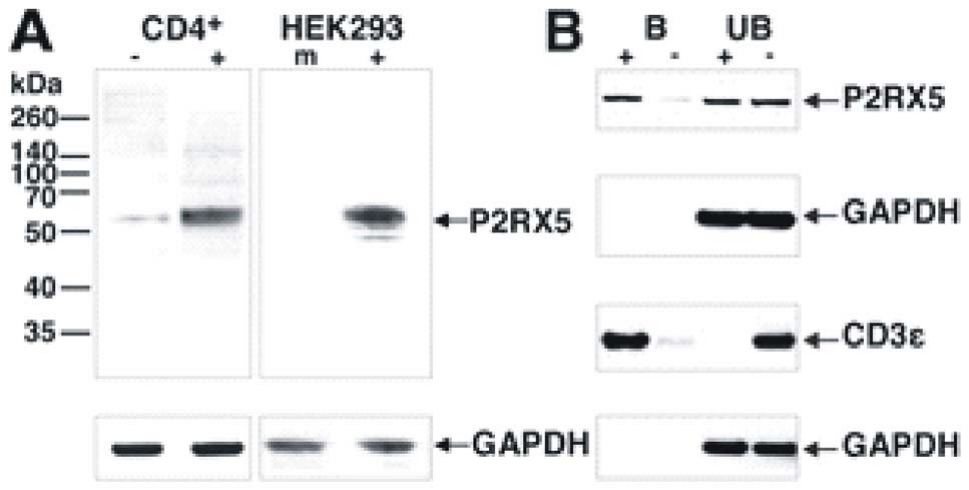
CD4^+^ T cell activation increases P2RX5 protein expression. A, Western blot of non-activated (CD4^+^/−) and activated (CD4^+^/+) CD4^+^ T cell lysates and of P2RX5 cDNA transfected (HEK293/+) or mock-transfected (HEK293/m) HEK293 cell lysates. Blots were stained with anti-P2RX5 antibody. Staining with anti-GAPDH antibody (arrow) served as loading control. B, Western blot of protein biotinylated at the cell surface of activated CD4^+^ T cells (+) and of controls (−). Protein was fractionated with streptavidin-agarose. **B** – streptavidin-agarose bound fraction; **UB** – unbound protein fraction. Western blots were stained with antibodies against P2RX5, GAPDH, and CD3ε as indicated by arrows.

The exponential time course of P2RX5 mRNA upregulation in stimulated CD4^+^ T cells (Fig. 1D) was well described with one time constant (τ = 19.5 hr). In comparison, CD25 mRNA upregulation, which occurs early in T cell activation [15], is significantly faster (τ = 7.4 hr) and quite insensitive to cycloheximide, a protein synthesis inhibitor (Fig. S1C). By contrast, stimulation of P2RX5 mRNA expression was highly sensitive to cycloheximide (Fig. 1D). Using a monoclonal antibody raised against aa 126 to 224, Western blot analysis of P2RX5 expression showed that activated CD4^+^ T cells express significantly more protein than resting CD4^+^ T cells (Fig. 2A). Importantly, HEK293 cells transfected with truncated P2RX5 cDNA show a distinct signal at 55–60 kDa which correlates with the T cell-derived signal. A comparably weak signal can be detected at 45–50 kDa which corresponds to unmodified truncated P2RX5 protein. The increase in P2RX5 protein expression matches the observed increase in P2RX5 mRNA expression.

### P2RX5 is recruited to the surface of activated CD4^+^ T cells

Next, we investigated the membrane localization of P2RX5 in soluble anti-CD3 antibody-activated CD4^+^ T cells. Using sulfo-NHS-SS-biotin to biotinylate cell surface proteins, we separated biotinylated and non-biotinylated protein for Western blot analysis (Fig. 2). We used CD3ε and GAPDH as a marker for cell surface and cytoplasmic protein, respectively. Controls showed that P2RX5 protein appeared in lysates of resting CD4^+^ T cells exclusively in the non-biotinylated protein fraction (UB- in Fig. 2B). In lysates of activated CD4^+^ T cells, however, we detected P2RX5 in both the biotinylated and the non-biotinylated protein fractions (Fig. 2B). Clearly, P2RX5 protein is able to localize to the cell surface of activated human CD4^+^ T cells.

**Figure 3:**
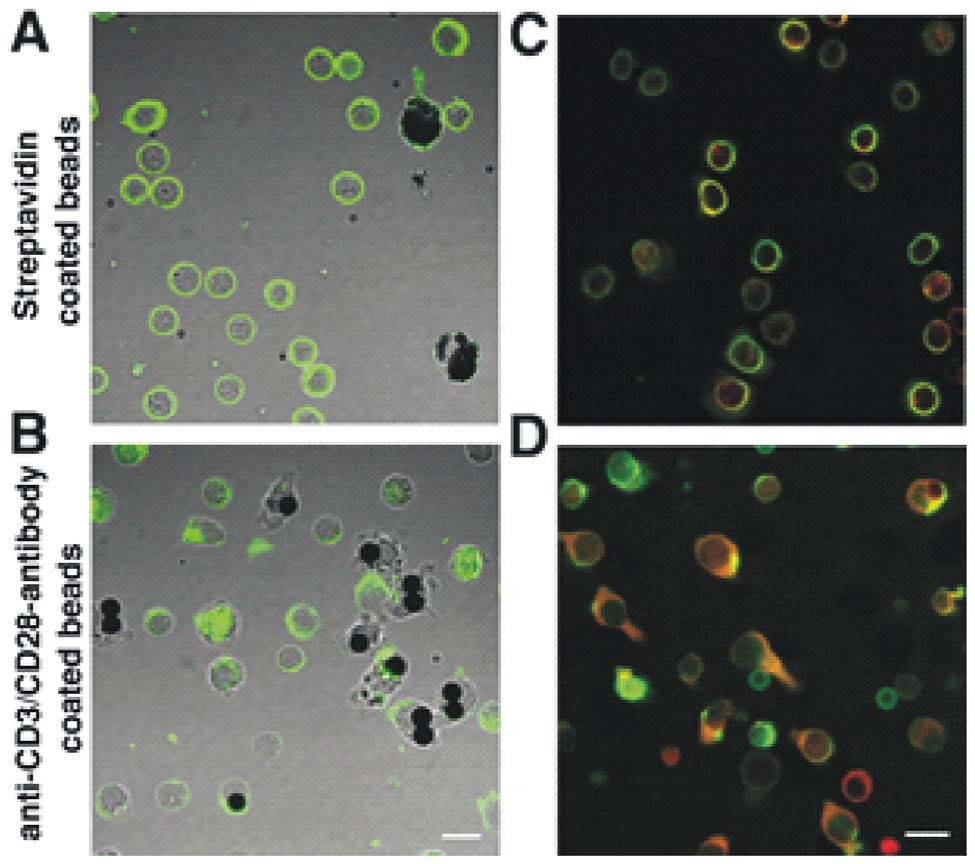
P2RX5 protein is redistributed upon T cell stimulation. T cells were incubated with either streptavidin beads for control (A, C) or anti-CD3/CD28 antibody-coated streptavidin beads (B, D) for activation. A, B, immunostaining pattern obtained with anti-P2RX5 antibodies (green). C, D, overlayed double fluorescence images obtained with anti-talin (green) and with anti-P2RX5 antibodies (red), pictures represent magnifications of overviews depicted in Fig. S1D. Scale bars – 10 µm.

In agreement with the biochemical data, immunofluorescent staining experiments using polyclonal anti-P2RX5 antibody showed that resting CD4^+^ T cells display a homogeneous distribution of P2RX5 protein at and below the cell surface (Fig. 3A). In activated CD4^+^ T cells, by contrast, P2RX5 protein exhibited a more polar distribution (Fig. 3B). Double-image immunofluorescence analysis of activated CD4^+^ T cells demonstrated that talin, a typical SMAC protein, and P2RX5 colocalize both in resting and in activated CD4^+^ T cells (Fig. 3C, D; Fig. S1D). The results suggest that P2RX5 is not only recruited to the membrane of activated T cells, but it may also be involved in T cell polarity upon activation.

### P2RX5 plays a role in sustaining CD4^+^ T cell polarity

**Figure 4:**
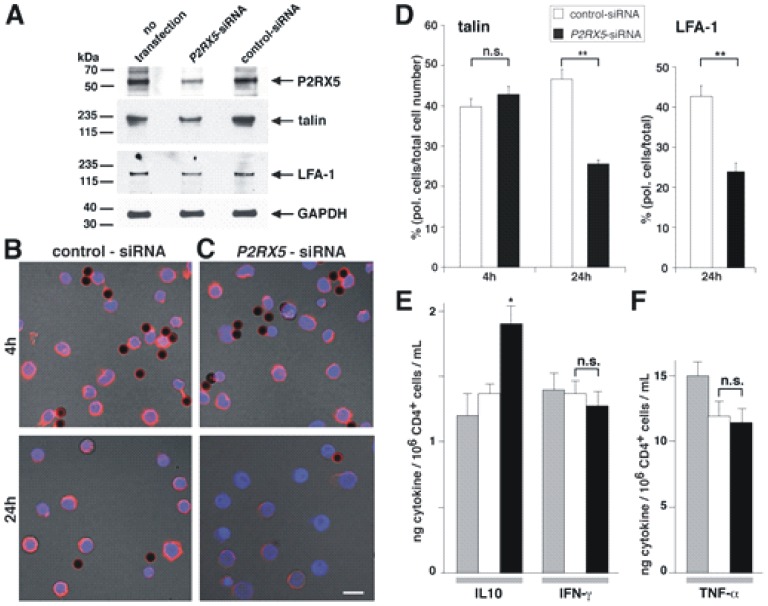
P2RX5 knock-down affects CD4^+^ T cell polarity and IL-10 secretion. A, Western blot analysis of P2RX5, talin, and LFA-1 expression in activated CD4^+^ T cells transfected with siRNA as indicated. GAPDH served as loading control. B, C, CD4^+^ T cells were transfected with indicated siRNA followed by activation for 4 or 24 h. Cells were stained with anti-talin antibodies. Scale bar – 10 µm. D, Bar diagram illustrating relative number of polarized CD4^+^ T cells seen with talin or LFA-1 immunostains. E, Bar diagram illustrating interleukin production of activated non-transfected (grey bars), control siRNA (white bars) or P2RX5-siRNA transfected (black bars) CD4^+^ T cells (n = 3 donors). Error bars are SEM, n.s. – non significant; * - p<0.05, ** p<0.01 (Student's T-test).

SMAC proteins are involved in IS establishment, organization, and maintenance [3]. Based on the above data we reasoned that P2RX5 participates in later stages of SMAC formation and/or maintenance as well as subsequent steps following T cell activation. This hypothesis was addressed in knock-down experiments. CD4^+^ T cells were transfected with siRNA directed against P2RX5 mRNA (P2RX5-siRNA) in order to interfere with P2RX5 expression. Scrambled siRNA served as control (control-siRNA). The transfection efficiency was ∼50 to 60% based on experiments with eGFP cDNA (data not shown). Western blot analysis of activated, P2RX5-siRNA-transfected CD4^+^ T cells indicated an approximately 50% reduction in P2RX5 protein expression (Fig. 4A). Next, we investigated the effect of siRNA transfection on CD4^+^ T cell polarity with talin as SMAC marker. 4 h after activation, we observed no significant difference in the polar distribution of talin between P2RX5-siRNA-transfected CD4^+^ T cells and controls (Fig. 4B), indicating that P2RX5 is not involved in the initial establishment of CD4^+^ T cell polarity. In contrast, 24 h after activation, P2RX5-siRNA-transfected CD4^+^-cells exhibited a significant loss of polarized talin distribution concomitant with a markedly reduced intensity of talin immunostain (Fig. 4C). Quantitative analysis of the immunostaining patterns (Fig. 4D) showed approximately half as many CD4^+^ T cells with a polarized talin distribution in P2RX5-siRNA- versus in control-siRNA-transfected cells (Fig. 4D). We obtained very similar data, when we immunostained siRNA-transfected and activated CD4^+^ T cells with anti-LFA-1 antibodies (Fig. 4D). Our results emphasize the involvement of P2RX5 in sustained polar SMAC protein distribution in activated CD4^+^ T cells.

### P2RX5 modulates IL-10 production of CD4^+^ T cells

The effect of P2RX5 on CD4^+^ T cell polarity resembled that of class-I MHC-restricted T cell-associated molecule (Crtam), a transmembrane protein of the Ig superfamily. Importantly, Crtam enables activated CD4^+^Crtam^+^ T cells to selectively produce more IFN-γ and IL-22 [4]. The comparison of P2RX5 and Crtam properties suggested that P2RX5 might also modulate the cytokine production of activated CD4^+^ T cells. Thus, we assessed the production of IL-1β, IL-2, IL-4, IL-5, IL-6, IL-8, IL-10, and IL-12p70, IFN-γ, TNF-α, and TNF-β assaying non-transfected-, P2RX5-siRNA-transfected, and control-siRNA-transfected CD4^+^ T cells, respectively. CD4^+^ T cells activated with anti-CD3/CD28 antibody-coated beads mainly produced IL-2 (Fig. S1E). In addition, we detected significant amounts of IL-10, IFN-γ, and TNF-α (Fig. 4E, F). Concentrations of other interleukins were very low or below the detection limit (Fig. S1E). Interestingly, we observed that activated P2RX5-siRNA-transfected CD4^+^ T cells produced significantly more IL-10 than controls, although the knock-down was incomplete (Fig. 4E). Production of all other interleukins that we tested was unaffected by the P2RX5 knock-down. Taken together, our data indicate that P2RX5 among the examined cytokines selectively modulates the production of IL-10 at a later phase of CD4^+^ T cell activation.

The comparison of P2RX5 and Crtam showed another interesting difference. Crtam^−^ T cells are hyper-proliferative in comparison to controls. In contrast, knock-down of P2RX5 leads to a reduction in T cell number. In comparison to controls, we noted a significant reduction (∼60%) in the number of activated CD4^+^ T cells after transfection with P2RX5-siRNA (Fig. S1F).

### P2RX5 expression in T cell subsets

We analyzed the surface P2RX5 expression in bulk CD4^+^ and CD8^+^ T cells as well as their respective CD45RA^+^CD27^+^ naïve and CD45RA^-^CD27^+^ memory subsets (Fig. S1G, H). Our data indicate that only a fraction of resting CD4^+^ and CD8^+^ T cells expresses P2RX5, and that this fraction is almost three times higher in CD8^+^ T cells. Furthermore, in both T cell compartments P2RX5 expression is higher in the resting naïve population albeit only slightly in CD4^+^ and clearly more pronounced in naïve CD8^+^ T cells.

### CD4^+^ T cell clones upregulate expression of P2RX5 in response to stimulation

**Figure 5:**
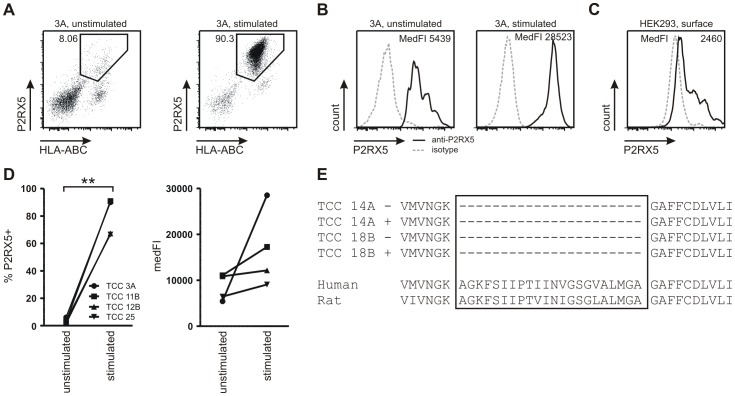
Expression of P2RX5 by human T cells is highly dynamic and activation-dependent. A, Stimulation of human CD4^+^ TCCs resulted in a significant increase in frequencies of cells expressing P2RX5 on the cell surface. B, Notably, surface expression levels were also upregulated. C, P2RX5 expression was considerably lower on the surface of HEK293 cells. D, Summary of significantly increased frequencies of surface P2RX5-expressing TCCs upon stimulation. Cell surface expression of P2RX5 was also upregulated on CD4^+^ TCCs. Bars represent mean values ±SEM. Statistical analysis was performed by paired t-test, **p<0.01. E, Sequencing of two CD4^+^ TCCs cultivated under stimulated (+) or unstimulated (−) conditions revealed that human Th1 and Th1/2 cells were expressing a transcript variant of P2RX5 lacking exon 10.

The above described differences of P2RX5 expression in CD4^+^ versus CD8^+^ T cells and their differentiation stages rendered interpretation of the dynamics difficult, and therefore we next examined the expression of P2RX5 in well-defined antigen-specific CD4^+^ TCCs [23]. We utilized two IFN-γ-secreting Th1 TCCs designated 3A and 11B, one IFN-γ and IL-17 double-producing TCC 25 as well as one TCC 12B, which produced IFN-γ but also IL-4 representing a Th1-2 phenotype (Table S3). Regardless of the phenotype we found a strong increase in frequencies of P2RX5^+^ cells in cell surface staining experiments if TCCs were stimulated (Fig. 5A). Moreover, also in the case of intracellular stainings the P2RX5^+^ populations of TCCs were markedly increased upon stimulation (data not shown). This highly dynamic, increased frequency of cells expressing P2RX5 was also accompanied by an increase of P2RX5 expression levels on the cell surface (Fig. 5B). As a control, we cultured HEK293 cells with or without beads coated with antibodies against CD2, CD3 and CD28. After 24 h we subjected these cells to surface as well as intracellular staining for P2RX5. Regardless of the culture conditions HEK293 cells were found to be positive for P2RX5. When focusing on expression levels, only a limited number of P2RX5 was found to be located on the cell surface of HEK293 cells (medFI  = 2,460, Fig. 5C) whereas these cells contained high intracellular amounts of P2RX5 (medFI  = 42,377) consistent with published data [24]. In contrast to HEK293 cells, P2RX5 expression by TCCs appeared to be induced upon activation. The strong intracellular, but also cell surface upregulation resulted in significantly elevated frequencies of P2RX5^+^ TCCs (Fig. 5D). The data illustrate the tight regulation of P2RX5 expression by activation in T cells.

### TCCs express a spliced variant of P2RX5

Th1 TCC 18B as well as TCC 14A showing a Th1-2 phenotype were used to address if P2RX5 expressed by T cells is the full-length molecule or the previously described truncation variant. mRNA was isolated and PCR-amplified after 24 h of cell culture with or without activation by anti-CD3. Sequencing revealed that human TCCs expressed a splice variant, which lacks exon 10 comprising 66 nucleotides (Fig. 5E). These results confirmed our previous findings, which were generated by culture of enriched CD4^+^ T cells with or without the addition of anti-CD3 (data not shown). Thus, human T cells do not express the full-length P2RX5, which functions as an ion channel.

## Discussion

Our work reveals a novel P2RX5 function in human immune cell activation. Full length P2RX5 expresses an established purinergic cation channel [25]–[29]. Here we show a link between the activation of human T cells and the expression of a P2RX5 variant, which contains an internal deletion disabling P2RX5 channel function [24], [30]. The truncated P2RX5 isoform serves as an “activation marker” molecule of T cells upon stimulation. Furthermore, it acts as a modulator of IL-10 production.

Starting from a systematic analysis of ion channel genes that are expressed upon T cell activation, we observed the most prominent increase for P2RX5 mRNA and therefore focused on this molecule. Subsequent studies of its expression time-course and cycloheximide sensitivity in activated CD4^+^ T cells argue against an important role of P2RX5 protein in the initial phase of CD4^+^ T cell activation. The observed increase in P2RX5 mRNA and protein concentration is likely a secondary or delayed response and suggests a functional role of P2RX5 in a later phase of CD4^+^ T cell activation.

Human CD4^+^ T cells express a P2RX5 variant (P2RX5Δ) lacking part of the second membrane-spanning segment that is essential for formation of the conduction pathway and P2R-ion channel activity [24], [30]. It is unlikely that lymphoid P2RX5Δ protein participates in P2R-like cation channel assembly [20], [24]. Previous heterologous expression studies in tissue culture cells even suggested that P2RX5Δ does not migrate to the cell surface [24]. Here, we show that P2RX5Δ protein is able to localize to the cell surface of activated human CD4^+^ T cells. The polar distribution of P2RX5Δ resembles that of CRAC and KCNN4 channels, which accumulate shortly after activation within an IS-associated SMAC [19]. Indeed, colocalization with talin suggests that P2RX5Δ represents a novel SMAC protein component.

The trimeric architecture of functional P2RX5 channels depends on the presence of Asp^355^ within transmembrane 2 (TM2) region of P2RX5 [24]. The deletion of a significant part of TM2 in P2RX5Δ most likely obviates P2RX5 trimerization into functional channels [30]. Due to the TM2 deletion, P2RX5Δ may adopt a membrane topology with only one transmembrane spanning segment (TM1) and a carboxy-terminus pointing towards the extracellular space, if P2RX5Δ is transported to the cell surface, like in activated CD4^+^ cells. Important to note, P2RX5Δ, when overexpressed in *in vitro* expression systems, has the tendency to aggregate [24]. An attractive hypothesis is that P2RX5Δ aggregate formation at the cell surface may play a role in homotypic or heterotypic immune cell interactions. This proposed function of P2RX5Δ in activated T cells awaits further investigation, possibly by the use of monoclonal antibodies specific for intracellular, transmembrane or extracellular domains of P2RX5 once these become available. Also, the SMAC localization of P2XR5 may indirectly influence CRAC channel activity and Ca^2+^ mobilization in lymphocytes, which should be investigated in the future.

By performing knock-down experiments of P2RX5 in activated CD4^+^ T cells we showed that P2RX5Δ affects the polar distribution of SMAC proteins talin and LFA-1 during a later phase of CD4^+^ T cell activation (between >4 h and <24 h). The consequence of P2RX5Δ expression on T cell polarity indicates mechanistic similarities with Crtam. Crtam is essential for sustaining CD4^+^ T cell polarity at a later phase of T cell activation in a subset of CD4^+^ T cells (CD4^+^Crtam^+^ T cells). Crtam is not expressed on the surface of resting naïve CD4^+^CD62L^+^ T cells, but is upregulated on CD4^+^ T cell subsets 14 h after activation followed by down-modulation within 24 h, thereby modulating IFN-γ production [4]. Remarkably, our results also indicate a role for P2RX5Δ in the modulation of interleukin production by activated CD4^+^ T cells. Partial knock-down of P2RX5Δ resulted in a significant increase in IL-10 production upon T cell activation. The data indicates that P2RX5Δ and Crtam regulate the production of different cytokines. Possibly, P2RX5Δ and Crtam are active in different subsets of CD4^+^ T cells or alternatively in the case of P2RX5Δ the relative expression level influences cytokine secretion and subsequently functional outcomes of T cell/APC interaction. Since none of the available commercial anti-P2RX5 antibodies specifically recognized an external P2RX5Δ epitope, we were unable to test this hypothesis directly in respective cell sorting experiments.

IL-10 is a potent immunoregulatory and anti-inflammatory cytokine that may directly or indirectly inhibit T cell proliferation [31], [32]. Notably, organ-specific IL-10 production has been identified to evoke autoimmunity in a mouse model of diabetes [33]. In this context it is noteworthy that a recent report identified hematopoietic-restricted minor histocompatibility antigen, LRH-1, as a P2RX5 splice variant with an altered C-terminal sequence. Aberrant expression of this splice variant leads to cytotoxic T cell-mediated cell lysis [34]. Also, a truncated P2RX5 protein resulting from a premature stop codon in the third exon of P2RX5 has been implicated in certain lymphoid malignancies [22]. It will be interesting to investigate if alterations of T cell activation and IL-10 production occur in these malignancies.

With respect to P2RX5^+^ fractions within CD4^+^ and CD8^+^ T cells we can only speculate about the possible causes at the moment. The higher frequency of P2RX5-expressing naïve versus P2RX5^+^ T cells in both compartments could indicate that P2RX5^+^ naïve cells recently received a stimulus, e.g. TCR-HLA/self-peptide interactions, which are the main signal for homeostatic proliferation. Interactions between costimulatory molecules such as CD80 or CD86 on APCs and their ligands (CD28/CTLA-4) influence the level of T cell activation [35]–[37]. Different costimulatory requirements of CD4^+^ and CD8^+^ T cell responses point to distinct mechanisms to activate these cells [38]–[40], and P2RX5 may be an additional factor involved in modulating T cell activation, however, dissecting the expression of P2RX5 in subsets of T cells, its role in their activation and possible effects on functional differentiation all need to be studied in the future.

Mechanistic changes of P2RX5Δ expression upon stimulation became even more evident in fully differentiated, clonal T cell populations. T cell activation increased the fraction of P2RX5^+^ cells within a TCC with respect to both surface and intracellular stainings as well as the level of surface P2RX5Δ expression in comparison to resting TCCs. Thus, the data obtained with antigen-specific TCCs confirmed the indicative expression patterns of P2RX5Δ in bulk populations as a function of activation of a T cell.

In summary our data demonstrate a role of P2RX5Δ, a truncated isoform of the P2RX5 ion channel in the late stage of T cell activation. They provide the basis for further studies of the role of this molecule in physiological and pathological conditions.

## Materials and Methods

### Ethics Statement

The protocol, under which the studies were pursued, was approved by the responsible local IRB, i.e. the Ethik Kommission der Ärztekammer Hamburg (Number of approved protocol: 2758). All participants signed a written informed consent prior to donating any samples, and the consent documents were part of the abovementioned, approved protocol. Consent documents are kept on file as part of the conduct of studies with human samples according to international standards and as stipulated by the responsible IRB.

### Cell Culture

PBMCs were isolated through a Ficoll (Biochrom, Berlin, Germany) density gradient centrifugation (400xg, 40 min, RT). PBMCs were resuspended in MACS buffer (0.5% BSA, 2.0 mM EDTA in PBS). PBMC activation was initiated by adding PHA-L (Sigma-Aldrich, St. Louis, MO, USA) to a final concentration of 31 nM for 72 h at 37°C. Where indicated, we activated by addition of 10 µg anti-CD3 rabbit antibodies/ml (DOKA, Glostrup, Denmark). CD4^+^ and CD8^+^ T cells were isolated by negative selection using the CD4^+^/CD8^+^ T cell Isolation Kit II (Miltenyi Biotec, Bergisch Gladbach, Germany) following the manufacturer's instructions. Purified T cells were resuspended in RPMI 1640 medium supplemented with 10% FCS (PAA, Pasching, Austria), 1% L-glutamine, 100 units/ml penicillin, and 0.1 mg/ml streptomycin (Invitrogen, Carlsbad, CA, USA). Purity of isolated CD4^+^ and CD8^+^ T cells was 94–97%. Stimulation of purified CD4^+^ and CD8^+^ T cells was performed in 96-well microtiter plates (Microtest Tissue Culture Plates, Falcon, BD Biosciences, Franklin Lakes, NJ, USA). Each well contained 2×10^5^ cells in 200 µL X-Vivo 15-medium (Lonza, Basel, Switzerland), to which 5×10^4^ anti-CD3/CD28 monoclonal antibody-coated beads (Invitrogen) were added. Unless indicated otherwise, incubation times were 72 h at 37°C. Experiments in the presence of cycloheximide were done adding 50 ng/ml to the medium for the times indicated.

### Microarray procedures

Total RNA was isolated from 1×10^7^ cells using TRIzol (Invitrogen). Fluorescent cDNA probes for stimulated and unstimulated samples were prepared by reverse transcription of mRNAs with aminoallyl-dUTP followed by Cy3 and Cy5 labelling according to the manufacturer's protocol (Amersham Pharmacia Biotech, Uppsala, Sweden). Labeled cDNAs were hybridized to oligonucleotide-based custom-made array. Array composition and evaluation is given in Tables S1, S2. Hybridization chambers were from Monterey Industries, Richmond, CA, USA. Blocking hybridization and staining was as described [41], [42]. Hybridization signals were scanned with ‘arrayWORX' scanner (Applied Precision, Issaquah, WA, USA). Fluorescent images of hybridized microarrays were analyzed with ScanAlyze software (http://rana.lbl.gov/EisenSoftware.htm). Fluorescence ratios were stored in a custom data base, and normalized data were extracted from this data base for further analysis with Array Assist software (Stratagene, La Jolla, CA, USA). Array data were filtered by selecting signals that were present on at least 75% of the arrays, had a spot diameter of ≥25 µm and a signal intensity of ≥200 in each channel. For normalization of hybridization signals, we added externally defined amounts of cDNA of three *Zea maize* genes (aeI, DD1B, CE11). Probes for α-IL-2 receptor (IL2RA or CD25) served as positive control for increased mRNA expression in activated PBMCs [43].

Affymetrix (Santa Clara, CA, USA) protocols were used for hybridization with labeled cDNA, washing and scanning of Affymetrix U133A 2.0 human genome arrays. Scanned microarray images were analyzed with Affymetrix Analysis Suite software with default analysis parameters. Data were then exported for querying and annotation (GDS785). Microarray data is available at www.ncbi.nlm.nih.gov/geo/ (GEO accession: GSE22387, GSE21837).

### Real-time quantitative polymerase chain reactions

RNA, extracted from PHA-L or anti-CD3 antibody (clone OKT3)-activated T cells with TRIzol reagent, was reverse transcribed with SuperScript First-Strand Synthesis system for RT-PCR (Invitrogen). For quantitative PCR (qPCR) we used prefabricated Taqman assays and a 7900 HT Sequence Detection System instrument (Applied Biosystems, Foster City, CA, USA). Data were normalized to CD247 gene expression to calculate n-fold expression changes.

### Transfection of HEK293 cells

HEK293 cells were transfected with P2RX5-pcDNA3 and lipofectamin following the manufacturer's protocol (Qiagen, Hilden, Germany). For control we transfected HEK293 cells with empty pcDNA3 vector.

### Biotinylation of cell surface protein

Soluble anti-CD3 antibody-activated CD4^+^ T cells were labeled with 10 mM sulfo-NHS-SS-biotin. Biotinylated cell surface proteins were separated from non-biotinylated intracellular proteins using the cell surface protein isolation kit (Pierce, Rockford, IL, USA). Collected fractions of bound and unbound protein were immunoblotted for analysis.

### Immunoblotting and immunocytochemistry

CD4^+^ T cells resuspended in 1% protein inhibitor mix (Sigma-Aldrich) were lysed by repeated freezing in liquid nitrogen and thawing. After adding 20× PBS lysates were spun at 2000xg for 3 min. The supernatant was centrifuged at 18,000xg for 20 min. The pellet was taken up in 100 mM NaCl, 50 mM HEPES (pH 7.4), 1% protein inhibitor mix, and 0.5% Triton-X-100 (Carl Roth, Karlsruhe, Germany). Proteins were separated on 10% NuPAGE Bis-Tris gels (Invitrogen) and blotted onto nitrocellulose membranes (Mini-Transblot, Bio-Rad, Munich, Germany) for immunodetection. Primary antibodies were anti-P2RX5 (clone 1C5 for immunoblotting or polyclonal for immunocytochemistry, Abnova, Taipei, Taiwan), anti-talin (clone 8d4, Sigma-Aldrich), anti-GAPDH (clone GAPDH-71.1, Sigma-Aldrich) monoclonal mouse antibodies, and anti-LFA-1 (against integrin, alpha-L, clone EP1285Y, Abcam, Cambridge, UK) monoclonal rabbit antibody. Secondary antibodies were anti-mouse HRP-coupled polyclonal sheep IgGs (Jackson ImmunoResearch, Newmarket, UK) and anti-rabbit HRP-coupled polyclonal IgGs (Jackson ImmunoResearch). CD4^+^ T cells were fixed and immunostained essentially as described [44]. Secondary Alexa488- and Alexa546-conjugated antibodies were from Invitrogen. Fluorescence images were taken with a Fluoview Fv1000 confocal microscope (Olympus, Tokio, Japan).

### siRNA transfection experiments

CD4^+^ T cells were transfected with P2RX5- and control-siRNA (Dharmacon, Lafayette, CO, USA, lot No. L-006286-00-0005 and, respectively, L-001810-10-05) using the Human T Cell Nucleofector Kit (Amaxa, Germany). Transfection efficiency was assessed under an inverted fluorescence microscope by counting cells with eGFP fluorescence versus total cell number. 12 h later anti-CD3/CD28 antibody-coated beads (Invitrogen) were added for activation.

### ELISAs for cytokine concentrations

Activated CD4^+^ T cells were sedimented 72 h after activation. The supernatant was harvested to determine concentrations of IL-1β, IL-2, IL-4, IL-5, IL-6, IL-8, IL-10, and IL-12p70, IFN-γ, TNF-α, and TNF-β using Quantikine Mouse Immunoassay according to the manufacturer's protocol (R&D Systems, Minneapolis, MN, USA). Data were read out using an LSR II flow cytometer (BD Biosciences) following the manufacturer's protocol. Data were evaluated with Bender Med Systems software (http://www.bendermedsystems.com/software-flowcytomix-pro—51).

### Cell culture of TCCs for P2RX5 analysis by flow cytometry and sequencing

TCCs were grown at 2×10^5^ cells/well in 96-well plates. TCC characteristics are summarized (Table S3). For sequencing, TCCs were grown in 48-well plates at 3×10^6^ cells/well. Cells were incubated for 24 h in X-Vivo 15 cell culture medium supplemented with or without beads coated with antibodies against CD2, CD3 and CD28 to stimulate cells according to the manufacturer's instructions (MACS T cell activation/expansion kit, Miltenyi). TCCs were characterized as mentioned elsewhere [23].

### Flow cytometry

Cells were stained for the exclusion of dead cells (LIVE/DEAD Fixable Dead Cell Stain Kit, Invitrogen). For surface staining cells were resuspended in blocking buffer (PBS, 10% donkey serum, 10% of 1 µg/ml human IgG (Jackson ImmunoResearch). After 15 min of incubation at 4°C, cells were stained using monoclonal antibodies against surface markers. Anti-CD3 Pacific Blue (clone UCHT1), anti-CD3 PE cyanine 7 (clone UCHT1), anti-CD4 allophycocyanin (APC) (clone RPA-T4), and anti-HLA-ABC PE (clone W6/32) antibodies were purchased from eBioscience (San Diego, CA, USA), anti-CD8 Pacific Blue (clone DN25) from Dako, and antibodies anti-CD27 APC Alexa750 (clone CLB-27/1), CD45RA PE cyanine 5.5 (clone MEM-56) from Invitrogen. In addition the polyclonal rabbit anti-human P2RX5 antibody (ProteinTech Group, Chicago, IL, USA) and the polyclonal rabbit IgG isotype control (Dianova, Hamburg, Germany) were included. Cells were stained for 30 min at 4°C. For detection of anti-P2RX5 antibody, cells were stained for 30 min at 4°C in the presence of donkey anti-rabbit IgG DyLight488 antibody (Jackson ImmunoResearch). Cells were washed with PBS, fixed and analyzed by flow cytometry.

For intracellular staining cells were fixed for 20 min at RT by the addition of Fixation Buffer (eBioscience). Cells were washed in permeabilization buffer (PBS, 5% FCS, 1 g/l Triton-X-100, 20 g/l BSA). Subsequently, cells were resuspended in blocking buffer which contained permeabilization buffer, 10% donkey serum as well as 10% of 1 µg/ml human IgG. After 15 min of incubation on ice the anti-CD3 Pacific Blue, anti-CD4 APC, anti-HLA-ABC PE, anti-P2RX5 antibodies and rabbit IgG isotype control antibodies were added, respectively. Cells were incubated for 30 min at 4°C and washed twice with permeabilization buffer. Afterwards anti-rabbit IgG DyLight488 antibody was added and cells were incubated for 30 min at 4°C. Cells were analyzed using a LSR II flow cytometer. Data was analyzed by using the FlowJo software (TreeStar, Ashland, OR, USA).

### Sequencing

TCCs were cultured for 24 h, centrifuged (5 min, 350xg), the cell pellets were resuspended in 350 µl buffer RLT (Qiagen) and frozen at -80°C. For RNA extraction, the RNeasy mini kit was used (Qiagen). RNA concentrations were determined by using the NanoDrop 1000 (Thermo Scientific, Waltham, MA, USA). 1 µg RNA of each approach was subjected to cDNA synthesis by following the instructions of the RevertAid H Minus First Strand cDNA Synthesis Kit (Thermo Scientific). Ten-fold dilutions of oligo (dT)-primed cDNA were amplified by PCR for 35 cycles of 45 sec at 95°C, 1 min at 60°C and 1.5 min at 72°C. Afterwards nested PCR was done. Primer sequences were: sense 5′-TCCCCAAATTCAACTTCTCC-3′, antisense 5′-TTCTGACTGCTGCTTCCACG-3′, sense (nested) 5′-GCAATGTGATGGAC-GTCAAGG-3′ and antisense (nested) 5′-TGAGCTGCTCAGATAGCCCC-3′. The PCR products were loaded on a 1.5% agarose gel for electrophoresis. Bands of approximately 516 bp were eluted using the Gel-Extraction Kit (GE Healthcare, Chalfont St Giles, UK). Finally, PCR products were used as templates in a second nested PCR, seperated on a 1.5% agarose gel and extracted as described before. The final PCR product was used for sequencing with a BigDye Terminator V1.1 cycle sequencing kit (Applied Biosystems) and a 3130 genetic analyzer (Applied Biosystems/Hitachi).

## Supporting Information

Figure S1 **Expression of P2RX5 by human T cells is activation-dependent.** A, KCNN4 and P2RX5 mRNA expression changes were compared in CD4^+^ and CD8^+^ T cells activated with different protocols. Activation protocols are indicated at the right-hand side. PBMCs were activated with PHA-L in medium with (+) or without (−) serum (X-Vivo 15, Lonza), or with anti-CD3 antibodies (DOKA) in serum free medium. CD4^+^ and CD8^+^ T cells were separated after activation and subjected to mRNA expression analysis by qPCR (see Materials and Methods). Error bars are SEM, n = 3. n.s. – not significant; _*_ - significant. Significance was analyzed using two-way ANOVA test (p<0.0001), Bonferroni-Posthoc test, and Students T-Test (p<0.05). B, Ion channel mRNA expression changed upon activation of PBMCs with anti-CD3 antibody. Experimental conditions were as described above. Error bars are SEM, n = 3. C, CD25 mRNA expression increased in activated CD4^+^ T cells in the course of time. CD4^+^ T cells were activated with anti-CD3/CD28 antibody-coated beads. CD25 mRNA expression level (□) was determined with qPCRs at the times indicated (n = 3, SEM). Δ – mRNA expression level after 12 h in the presence of cycloheximide. For further details see Materials and Methods. D, P2RX5 protein colocalized with talin in the IS. CD4^+^ T cells were incubated with either streptavidin beads for control or anti-CD3/CD28 antibody-coated streptavidin beads for activation. Overlay of staining patterns obtained with anti-talin (green) and anti-P2RX5 antibodies (red), pictures represent overviews of magnifications shown in Fig. 3. Scale bars – 10 µm. E, Activated CD4^+^ T cells transfected with P2RX5 siRNA or control siRNA produced interleukins. CD4^+^ T cells transfected with P2RX5-siRNA or control-siRNA were activated for 72 h with anti-CD3/CD28-coated beads. Subsequently, interleukin concentration was assessed in the supernatant by ELISA. F, Knock-down of P2RX5 mRNA decreased the number of activated CD4^+^ T cells. CD4^+^ T cells (5×10^6^ cells) were activated with anti-CD3/CD28 antibody-coated beads for 72 h. Subsequently cells were counted in a counting chamber. Cell numbers were normalized to those of untransfected control CD4^+^ T cells (gray bar; set to 100%). White and black bar - number of cells transfected with control-siRNA and P2RX5-siRNA, respectively. Error bars are SEM, n = 3. n.s. – not significant; _*_ - significant, Students T-test (p<0.005). G, PBMCs were used for analysis of P2RX5 expression by T cells. P2RX5^+^ subsets were identified in flow cytometry experiments in bulk CD4^+^ T cell populations, but also in naïve and memory CD4^+^ T cell subsets. In brief, after dead cell exclusion gated CD3^+^ HLA-ABC^+^ populations were used to further determine CD4^+^P2RX5^+^ T cell frequencies and medFI values. CD4^+^ T cells were also used to gate on CD45RA^+^CD27^+^ naïve and CD45RA^-^CD27^+^ memory CD4^+^ T cells for P2RX5 expression analysis. H, P2RX5 is expressed by a minor frequency of unstimulated CD4^+^ and CD8^+^ T cells of bulk PBMCs. Furthermore, the data indicate a higher frequency of P2RX5^+^ naïve T cells compared to P2RX5^+^ memory T cells. Bars represent mean values ±SEM (n = 3). Statistical analysis was performed by paired t-test.
(TIF)

Table S1 List of ion channel subunits probed on custom-made oligonucleotide array.
(DOCX)

Table S2 Changes in ion channel mRNA expression upon PBMC stimulation with PHA-L.
(DOCX)

Table S3 Characteristics of human TCCs used for P2RX5 protein expression analysis and RNA sequencing.
(DOCX)
