# Supplementary figures and images for: A Truncation Variant of the Cation Channel P2RX5 Is Upregulated during T Cell Activation
Source: PLoS One. 2014 Sep 2;9(9):e104692. doi: 10.1371/journal.pone.0104692 (PMC4152149; doi:10.1371/journal.pone.0104692)

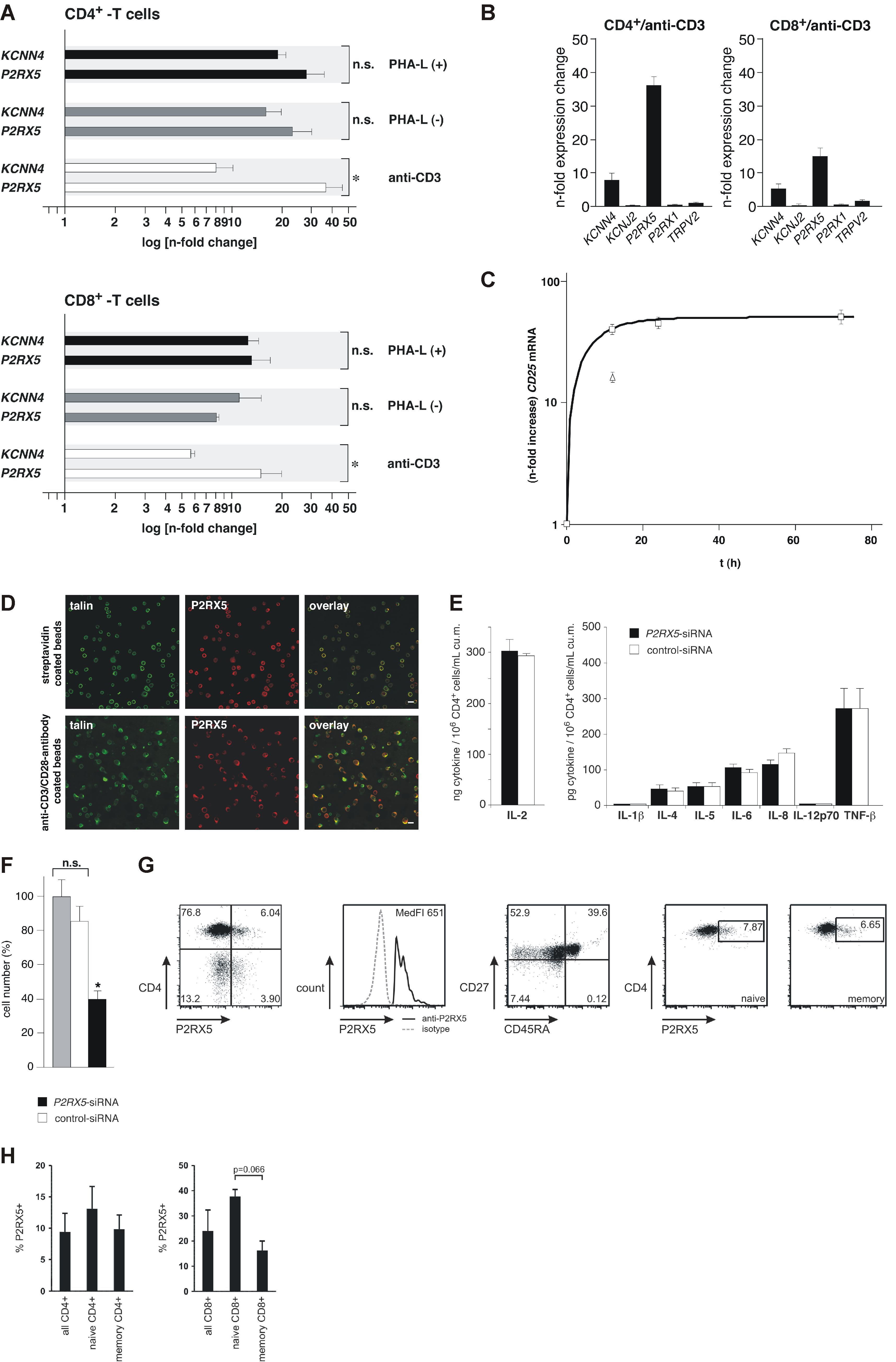

Supplement: Figure S1 — Expression of P2RX5 by human T cells is activation-dependent. A, KCNN4 and P2RX5 mRNA expression changes were compared in CD4+ and CD8+ T cells activated with different protocols. Activation protocols are indicated at the right-hand side. PBMCs were activated with PHA-L in medium with (+) or without (−) serum (X-Vivo 15, Lonza), or with anti-CD3 antibodies (DOKA) in serum free medium. CD4+ and CD8+ T cells were separated after activation and subjected to mRNA expression analysis by qPCR (see Materials and Methods). Error bars are SEM, n = 3. n.s. – not significant; * - significant. Significance was analyzed using two-way ANOVA test (p<0.0001), Bonferroni-Posthoc test, and Students T-Test (p<0.05). B, Ion channel mRNA expression changed upon activation of PBMCs with anti-CD3 antibody. Experimental conditions were as described above. Error bars are SEM, n = 3. C, CD25 mRNA expression increased in activated CD4+ T cells in the course of time. CD4+ T cells were activated with anti-CD3/CD28 antibody-coated beads. CD25 mRNA expression level (□) was determined with qPCRs at the times indicated (n = 3, SEM). Δ – mRNA expression level after 12 h in the presence of cycloheximide. For further details see Materials and Methods. D, P2RX5 protein colocalized with talin in the IS. CD4+ T cells were incubated with either streptavidin beads for control or anti-CD3/CD28 antibody-coated streptavidin beads for activation. Overlay of staining patterns obtained with anti-talin (green) and anti-P2RX5 antibodies (red), pictures represent overviews of magnifications shown in Fig. 3. Scale bars – 10 µm. E, Activated CD4+ T cells transfected with P2RX5 siRNA or control siRNA produced interleukins. CD4+ T cells transfected with P2RX5-siRNA or control-siRNA were activated for 72 h with anti-CD3/CD28-coated beads. Subsequently, interleukin concentration was assessed in the supernatant by ELISA. F, Knock-down of P2RX5 mRNA decreased the number of activated CD4+ T cells. CD4+ T cells (5×10^6^ cells) [file pone.0104692.s001.tif]
