# Supplementary material for: A Truncation Variant of the Cation Channel P2RX5 Is Upregulated during T Cell Activation
Source: PLoS One. 2014 Sep 2;9(9):e104692. doi: 10.1371/journal.pone.0104692 (PMC4152149; doi:10.1371/journal.pone.0104692)
Supplement: Table S1 — List of ion channel subunits probed on custom-made oligonucleotide array. (DOCX) [file pone.0104692.s002.docx]

**Supplemental Table S1**

List of ion channel subunits probed on custom-made oligonucleotide array.

**Calcium channel subunits**

| *CACNA1A* | *CACNA1D* | *CACNA1G* | *CACNA1S* | *CACNA2D3* | *CACNB2* | *CACNG1* | *CACNG4* | *CACNG7* |
| --- | --- | --- | --- | --- | --- | --- | --- | --- |
| *CACNA1B* | *CACNA1E* | *CACNA1H* | *CACNA2D1* | *CACNA2D4* | *CACNB3* | *CACNG2* | *CACNG5* | *CACNG8* |
| *CACNA1C* | *CACNA1F* | *CACNA1I* | *CACNA2D2* | *CACNB1* | *CACNB4* | *CACNG3* | *CACNG6* |  |

**Sodium channel subunits**

| *SCN1A* | *SCN2B* | *SCN4A* | *SCN5A* | *SCN8A* | *SCN10A* | *SCN12A* | *SCNN1A* | *SCNN1D* |
| --- | --- | --- | --- | --- | --- | --- | --- | --- |
| *SCN1B* | *SCN3A* | *SCN4B* | *SCN7A* | *SCN9A* | *SCN11A* | *SCNM1* | *SCNN1B* | *SCNN1G* |
| *SCN2A2* | *SCN3B* |  |  |  |  |  |  |  |

**TRP channel subunits**

| *TRPA1* | *TRPC3* | *TRPC5* | *TRPM1* | *TRPM4* | *TRPM7* | *TRPV1* | *TRPV3* | *TRPV5* |
| --- | --- | --- | --- | --- | --- | --- | --- | --- |
| *TRPC1* | *TRPC4* | *TRPC6* | *TRPM2* | *TRPM5* | *TRPM8* | *TRPV2* | *TRPV4* | *TRPV6* |
| *TRPC2* | *TRPC4AP* | *TRPC7* | *TRPM3* | *TRPM6* |  |  |  |  |

**Potassium channel subunits**

| *DPPX* | *KCNAB2* | *KCNE1* | *KCNH2* | *KCNIP4* | *KCNJ12* | *KCNK7* | *KCNMB3* | *KCNRG* |
| --- | --- | --- | --- | --- | --- | --- | --- | --- |
| *KCMF1* | *KCNAB3* | *KCNE1L* | *KCNH2-* | *KCNJ1* | *KCNJ13* | *KCNK9* | *KCNMB4* | *KCNS1* |
| *KCNA1* | *KCNB1* | *KCNE2* | *USO* | *KCNJ2* | *KCNJ14* | *KCNK10* | *KCNN1* | *KCNS2* |
| *KCNA2* | *KCNB2* | *KCNE3* | *KCNH3* | *KCNJ3* | *KCNJ15* | *KCNK12* | *KCNN2* | *KCNS3* |
| *KCNA3* | *KCNC1* | *KCNE4* | *KCNH4* | *KCNJ4* | *KCNJ16* | *KCNK13* | *KCNN3* | *KCNT1* |
| *KCNA4* | *KCNC2* | *KCNF1* | *KCNH5* | *KCNJ5* | *KCNK1* | *KCNK15* | *KCNN4* | *KCNU1* |
| *KCNA5* | *KCNC3* | *KCNG1* | *KCNH6* | *KCNJ6* | *KCNK2* | *KCNK16* | *KCNQ1* | *KCNV1* |
| *KCNA6* | *KCNC4* | *KCNG2* | *KCNH7* | *KCNJ8* | *KCNK3* | *KCNK17* | *KCNQ2* | *KCNV2* |
| *KCNA7* | *KCND1* | *KCNG3* | *KCNH8* | *KCNJ9* | *KCNK4* | *KCNMA1* | *KCNQ3* | *KCR1* |
| *KCNA10* | *KCND2* | *KCNG4* | *KCNIP2* | *KCNJ10* | *KCNK5* | *KCNMB1* | *KCNQ4* |  |
| *KCNAB1* | *KCND3* | *KCNH1* | *KCNIP3* | *KCNJ11* | *KCNK6* | *KCNMB2* | *KCNQ5* |  |

**Chloride channel subunits**

| *CLCA1* | *CLCA3* | *CLCN1* | *CLCN3* | *CLCN5* | *CLCN7* | *CLCNKB* | *CLNS1A* | *CLNS1B* |
| --- | --- | --- | --- | --- | --- | --- | --- | --- |
| *CLCA2* | *CLCA4* | *CLCN2* | *CLCN4* | *CLCN6* | *CLCNKA* |  |  |  |

**Miscellaneous channel subunits**

| *STIM1* | *Orai1* | *HCN1* | *HCN2* | *HCN3* | *HCN4* |
| --- | --- | --- | --- | --- | --- |

**Positive controls Negative controls**

| *Amp* | *eGFP* | *Shaker* |
| --- | --- | --- |

| *CD3* | *CD25* | *CD69* | *TCR* |
| --- | --- | --- | --- |

**Spiking controls**

| *ae I* | *DD1B* | *CE11* |
| --- | --- | --- |

**Other controls**

| *GAPDH* | *PPIA* |
| --- | --- |
| *β-actin* | *RPLPO* |
| *B-2M* | *TFRC* |
| *GUSB* | *TBP* |
| *HPRT1* | *18s rRNA* |
| *PGK1* |  |

Ion channel nomenclature is from http://www.ncbi.nlm.nih.gov/omim. Oligonucleotides were designed and purchased from Operon for custom-made array and had similar melting temperatures. Sequences can be supplied on request. Oligonucleotides were spotted on poly-L lysine coated glass slides using a spotting robot (TECAN) and then crosslinked by UV irradiation. Each oligonucleotide occurred four times in each array.
