# Supplementary material for: A Truncation Variant of the Cation Channel P2RX5 Is Upregulated during T Cell Activation
Source: PLoS One. 2014 Sep 2;9(9):e104692. doi: 10.1371/journal.pone.0104692 (PMC4152149; doi:10.1371/journal.pone.0104692)
Supplement: Table S2 — Changes in ion channel mRNA expression upon PBMC stimulation with PHA-L. (DOCX) [file pone.0104692.s003.docx]

**Supplemental Table S2**

Changes in ion channel mRNA expression upon PBMC stimulation with PHA-L.

| **Controls** | **Expression Change** | **Ion Channel Subunits** | **Expression Change** |
| --- | --- | --- | --- |
| *Ae I* | 1,01 ± 0,03 | *CACNA1C* | 0,83 ± 0,16 |
| *CE 11* | 0,93 ± 0,05 | *CACNA1E* | 0,96 ± 0,15 |
| *DD1B* | 0,94 ± 0,07 | *SCN12A* | 0,91 ± 0,07 |
|  |  | *TRPC4AP* | 1,73 ± 0,27 |
| *CD25* | 26,80 ± 8,32 | *TRPM7* | 1,28 ± 0,23 |
| *CD3* | 2,81 ± 0,41 | *TRPV1* | 1,41 ± 0,28 |
| *CD69* | 0,43 ± 0,06 | *TRPV2* | 2,02 ± 0,30 |
| *TCR* | 1,35 ± 0,15 | *KCMF1* | 1,66 ± 0,20 |
| *GAPDH* | 1,65 ± 0,32 | *KCNA3* | 0,81 ± 0,16 |
|  |  | *KCNAB2* | 2,19 ± 0,39 |
|  |  | *KCNG3* | 1,16 ± 0,23 |
|  |  | *KCNJ2* | 0,29 ± 0,05 |
|  |  | *KCNMA1* | 2,02 ± 0,30 |
|  |  | *KCNMB1* | 0,39 ± 0,04 |
|  |  | *KCNN4* | 2,40 ± 0,43 |
|  |  | *KCNQ2* | 1,01 ± 0,15 |
|  |  | *CLCN3* | 1,56 ± 0,34 |
|  |  | *CLCN7* | 2,09 ± 0,32 |
|  |  | *CLNS1A* | 2,49 ± 0,38 |
|  |  | *CLNS1B* | 0,84 ± 0,08 |
|  |  | *STIM1* | 2,80 ± 0,41 |
|  |  | *Orai1* | 3,0 ± 0,48 |
|  |  | *HCN2* | 1,82 ± 0,48 |

Changes in mRNA expression refer to the ratio of normalized fluorescence signal in activated versus non-activated PMBC samples and are averages (n = 20 ± SEM) of fourteen custom-made and of six Affimetrix array analyses, respectively. Expression changes of ≥ 2 were scored as significant and used to construct the bar diagram in Figure 1A.
