# Supplementary material for: A Truncation Variant of the Cation Channel P2RX5 Is Upregulated during T Cell Activation
Source: PLoS One. 2014 Sep 2;9(9):e104692. doi: 10.1371/journal.pone.0104692 (PMC4152149; doi:10.1371/journal.pone.0104692)
Supplement: Table S3 — Characteristics of human TCCs used for P2RX5 protein expression analysis and RNA sequencing. (DOCX) [file pone.0104692.s004.docx]

**Supplemental Table S3**

Characteristics of human TCCs used for P2RX5 protein expression analysis
and RNA sequencing.

| *TCC #* | *Fine specificity* | *CD4/CD8* | *Th phenotype* | *Restriction* | *Source* |
| --- | --- | --- | --- | --- | --- |
| 3A | VP1 91-105 | CD4^+^ | Th1 | DR2A/DR2B/Other | Brain |
| 11B | VP1 143-157 | CD4^+^ | Th1 | DR2A/DR2B/Other | Brain |
| 12B | VP1 229-243 | CD4^+^ | Th1-2 | DQW6 | Brain |
| 14A | VP1 74-88 | CD4^+^ | Th1-2 | DR2A/DR2B | Brain |
| 18B | VP1 34-48 | CD4^+^ | Th1 | DQW6 | Brain |
| 25 | n/a | CD4^+^ | Th17 | n/a | CSF |

Summary of TCCs with Th1, Th1-2 and Th17 phenotype, respectively. The TCCs are specific for JC polyoma virus and have been isolated from a brain biopsy sample of a multiple sclerosis patient who developed progressive multi-focal leucoencephalopathy (PML) and PML immune reconstitution inflammatory syndrome (IRIS) under natalizumab therapy. TCCs 3A-18B are specific for different peptides of the JC polyoma virus protein VP1.
